# Effectiveness and Limitations of Endovenous Laser Ablation for Anterior Saphenous Vein Insufficiency: A Single-Center Retrospective Study

**DOI:** 10.3390/jcm15051733

**Published:** 2026-02-25

**Authors:** Eva Gruber, Merian Ranjbaryan, Bachar el Jamal, Syrus Karsai, Eike Sebastian Debus, Lars Müller

**Affiliations:** 1 Dermatologikum Hamburg GmbH, 20354 Hamburg, Germany; 2 Department of Dermatology, Allergology and Venerology, University Medical Center Schleswig-Holstein—Campus Lübeck (UKSH), 23538 Lübeck, Germany; 3 Department of Dermatology, University Medical Center Hamburg-Eppendorf, 20251 Hamburg, Germany; 4 Department of Vascular Medicine, University Heart and Vascular Center, University Medical Center Hamburg-Eppendorf, 20251 Hamburg, Germany

**Keywords:** anterior accessory saphenous vein, AASV, venous reflux, truncal vein, laser ablation, radiofrequency ablation, EVLT, thermal ablation, morbidity, sclerotherapy

## Abstract

**Background**: Anterior saphenous vein (ASV) incompetence represents the third most common form of truncal varicose veins, and evidence for endovenous laser ablation (EVLA) in this setting remains limited. **Methods**: We evaluated outcomes of EVLA in cases with dominant ASV insufficiency. All EVLA procedures performed by a single surgeon between April 2019 and December 2023 for primary ASV reflux (ASV-R) were compared with a cohort containing all EVLA treatments for great saphenous vein (GSV) insufficiency without ASV reflux from April to December 2019 (GSV-R). We used a 1470-nm diode laser with radially emitting fibers for the interventions. **Results**: We included 378 patients (mean age 49.5 years): 208 and 256 treated limbs in the ASV-R and GSV-R cohorts, respectively. Female patients were more frequent in the ASV-R cohort than in the GSV-R cohort (80.5% vs. 62.9%, *p* < 0.001). ASV-R cases exhibited concomitant GSV insufficiency in 54.3% of cases. Redo procedures due to initial treatment failure were more frequent in ASV-R (1.9% vs. 0%, *p* = 0.04). Over a mean follow-up period of 332 days, 16 recurrences occurred in the ASV-R cohort compared with 4 in the GSV-R cohort, corresponding to a significantly increased hazard of recurrence in ASV-R (HR 8.41, 95% CI 2.78–25.4). Rates of subsequent foam sclerotherapy (16.8% vs. 10.5%) and minor complications (5.3% vs. 4.3%) did not differ significantly between ASV-R and GSV-R, respectively. ASV-R cases without concomitant GSV reflux demonstrated a higher need for secondary sclerotherapy, compared to ASV-R cases with additional GSV insufficiency. **Conclusions**: Our findings suggest that EVLA for ASV insufficiency is technically more challenging and yields inferior outcomes than EVLA for GSV incompetence. These considerations should be taken into account during preoperative planning and patient counseling. Further prospective and comparative analyses are needed to better define the effectiveness of thermal ablation strategies in ASV insufficiency and to support patient-centered, individualized treatment decisions.

## 1. Introduction

Insufficiency of the great saphenous vein (GSV) and small saphenous vein (SSV) is effectively treated by endovenous thermal ablation (EVTA), a well-established and guideline-endorsed therapeutic modality [1]. In contrast, the optimal treatment for insufficiency of the anterior saphenous vein (ASV) is less clearly defined.

The ASV is increasingly recognized as a true truncal vein, characterized by its own fascial compartment and the presence of venous valves [2,3]. Reflux via the ASV is considered to be of comparable clinical significance to reflux in the GSV trunk. Moreover, observations suggest that superficial thrombosis may occur more frequently in the presence of ASV insufficiency [4,5]. Formerly regarded as an ‘accessory’ branch of the GSV, the ASV runs along the proximal thigh, lateral to the GSV, with a typical length of 5–20 cm, before joining the femoral vein [3]. Both its length and its confluence pattern exhibit considerable anatomical variability. In most patients, reflux due to incompetence at the saphenofemoral junction (SFJ) drains into the GSV. However, in up to 20% of cases, a competent preterminal GSV valve diverts reflux into the ASV and its tributaries, either anterolaterally or anteromedially. In the anteromedial configuration, a communicating branch to the GSV is frequently present and may be associated with segmental GSV insufficiency [3,6,7].

The pronounced anatomical heterogeneity complicates the technical performance of endovenous thermal ablation in cases of ASV insufficiency. The ASV—a frequent source of recurrent varicosities after prior GSV ablation—may present as a very short, straight venous segment, complicating ultrasound-guided puncture and catheterization [3,7,8,9,10,11]. In addition, frequent communication with complex tributary networks raises questions regarding optimal concomitant treatment strategies. Despite its clinical relevance, data regarding endovenous laser or radiofrequency ablation for ASV insufficiency remain limited [12,13,14,15].

Accordingly, the aim of this study was to evaluate the feasibility and effectiveness of endovenous laser ablation (EVLA) for ASV insufficiency using unselected data from routine clinical practice, and to compare these outcomes with those achieved after EVLA for GSV insufficiency.

## 2. Materials and Methods

### 2.1. Study Design

We conducted a retrospective analysis of all primary EVLA procedures performed by a single operator (LM) for reflux involving the SFJ and the ASV between May 2019 and December 2023. These cases were assigned to the ASV reflux cohort (ASV-R). As a comparison cohort, all primary EVLA procedures performed between May 2019 and December 2019 for GSV insufficiency with proximal reflux, without concomitant ASV reflux, were included (GSV-R). Cases with a history of prior intervention or surgery involving the GSV or ASV—therefore representing either a recurrence or a new manifestation in an immediately adjacent region—were excluded.

Cases were identified using the practice information system’s operative schedule. Clinical indications, procedural details and all available follow-up data through December 2024 were extracted. Data extraction was conducted between September and December 2025, and all datasets were fully anonymized at the time of extraction. Although the two cohorts were derived from different calendar periods, all procedures were performed by the same operator using an identical technique and standardized peri-procedural protocols. Importantly, both cohorts were assembled consecutively, without case selection, reflecting routine clinical practice. Follow-up assessments were conducted within the same clinical setting using consistent criteria for recurrence and reintervention.

The study was submitted to the Ethics Committee of the Hamburg Medical Association, which confirmed that formal ethical consultation or approval was not required due to the exclusive use of anonymized data (reference no: PV7252-4650_2-WF).

### 2.2. Preoperative Diagnostics

The indication for intervention was defined through shared decision-making with patients as part of the preoperative assessment. All included patients were classified as CEAP C2s or higher according to the clinical, etiological, anatomical, and pathophysiological classification. Duplex ultrasound examination was performed in standing position, with a reflux duration of >0.5 s defined as pathological. The primary reflux-conducting vein distal to the saphenofemoral junction was anatomically characterized, and its diameter in the groin region was measured.

### 2.3. Endovenous Interventions

All procedures were performed under sterile conditions in a dedicated procedure room. Mild sedation was administered upon patient request. EVLA was carried out using a 1470-nm diode laser and dual-ring radially emitting fiber catheters (Biolitec AG, Vienna, Austria). Depending on anatomical requirements, either 6-French catheters with a 600-µm core fiber or 4-French catheters with a 400-µm core fiber were used. Six-French catheters were introduced via a vascular sheath using the so called Seldinger technique, whereas 4-French catheters were inserted under ultrasound guidance using a 16-G peripheral venous catheter.

All ablations were performed under tumescent anesthesia (1000 mL normal saline, 50 mL 1% mepivacaine, and 8 mL 8.4% sodium bicarbonate). The power output was 8 W (1.3 mm fiber) or 10 W (1.9 mm fiber). The linear endovenous energy density (LEED) target was 50–100 joules per cm of vein segment, dependent of the vein diameter. As an approximation, an energy output of 10 J/cm was targeted for each millimeter of vein diameter. In most ASV-R cases, flush occlusion of both the ASV and GSV was pursued by separate cannulations, with the GSV ablated at the proximal thigh. In cases with additional GSV reflux, the GSV was ablated over a long distance, covering the entire insufficient section and always including the competent proximal section up to the SFJ. In a subset of ASV-R cases, only the ASV was ablated up to its confluence with the GSV, while the GSV was left untreated.

In cases of GSV insufficiency, the treatment goal was flush occlusion of the GSV. Occlusion of the non-refluxing ASV was also performed as a preventive measure when this appeared appropriate based on the anatomy [16,17]. Accompanying phlebectomies or foam sclerotherapy with polidocanol were performed as clinically indicated. All patients received a single intraoperative subcutaneous dose of low-molecular-weight heparin (LMWH) for thromboprophylaxis; additional doses could be prescribed based on individual risk stratification. Postoperatively, all puncture sites and incisions were covered with sterile wound dressings.

### 2.4. Postoperative Follow-Up and Adjunctive Treatments

Clinical and duplex ultrasound follow-up was recommended 10–14 days postoperatively. The follow-up included physical examination, inspection of puncture and incision sites, and duplex ultrasonography to confirm deep venous patency, assess occlusion of treated vein segments, and verify elimination of reflux.

Therapeutic failure was defined as persistence of reflux due to non-occlusion of the main reflux-conducting vein.

Patients were offered annual follow-up and advised to return earlier in case of persistent or recurrent symptoms. Residual varicose tributaries were initially observed for a period of up to three months. If regression remained insufficient, additional foam sclerotherapy with polidocanol was offered. Patients were censored at the time of their last documented clinical assessment if no recurrence had occurred. There was no predefined minimum follow-up duration, and no systematic differences in follow-up procedures between cohorts.

### 2.5. Data Analysis and Statistics

Baseline patient characteristics, treatment indications, and procedural details were extracted from electronic medical records. Primary endpoints were immediate post-procedural technical success and the incidence of complications. Secondary endpoints included the need for additional sclerotherapy of residual tributaries and the frequency and timing of recurrences requiring repeated thermal ablation.

Descriptive statistics are presented as means (±standard deviation) or medians (range), as appropriate. Categorical variables were compared using the chi-squared test, with Fisher’s exact test applied when expected cell counts were less than five. Continuous variables were analyzed using Welch’s *t*-test for normally distributed data and the Mann–Whitney U test for non-parametric data. All tests were two-sided, and a *p*-value < 0.05 was considered statistically significant.

Time-to-event outcomes were analyzed using Kaplan–Meier estimates and Cox proportional hazards regression. Given that the size of the ASV-R cohort was fixed by clinical availability and that recurrence events were infrequent, no formal a priori sample size calculation was performed. Accordingly, the analysis focused on estimation of effect size (hazard ratio with 95% confidence interval) rather than hypothesis testing. Log-rank testing was used for descriptive comparison only. Statistical analyses were performed using R statistical software (version 4.5.2).

## 3. Results

### 3.1. Included Patients and Baseline Characteristics

Overall, treatment data from 378 patients were included, with a mean age of 49.5 years. In the ASV-R cohort, data from 208 treated limbs in 185 individual patients were analyzed, whereas the GSV-R cohort comprised 256 treated limbs in 197 patients. Four of the patients with reflux on both legs had one limb assigned to the ASV-R group and the contralateral limb to the GSV-R group. Mean follow-up duration differed modestly between cohorts (ASV-R: 298.6 days; GSV-R: 359.5 days). However, time-to-event analysis inherently accounts for variable follow-up durations and censoring, allowing for unbiased estimation of relative recurrence risk under the assumption of comparable censoring mechanisms.

Baseline characteristics are summarized in Table 1. Notably, the proportion of female patients was significantly higher in the ASV-R cohort (*p* < 0.001). In addition, the mean age was significantly lower in the ASV-R cohort compared with the GSV-R cohort (*p* = 0.004), while the body mass index (BMI) was higher in this group (*p* = 0.002). Regarding the clinical classification according to the CEAP criteria, the proportion of C2 cases was higher in the ASV-R group compared with patients with more advanced stages of chronic venous insufficiency (*p* < 0.001). The cases from 2019 had already been analyzed as part of a previous study [16]. Based on the reflux pattern, 22 treatments from this period were assigned to the ASV-R group, and 256 treatments constituted the GSV-R group.

### 3.2. Results of Ablation Treatments

In both cohorts, the fundamental ablation strategy was largely identical. The objective was flush occlusion of the truncal vein primarily responsible for axial reflux, as well as occlusion of the respective non-refluxive truncal vein [16]. In the majority of ASV-R treatments, the proximal GSV was also treated via a separate puncture and cannulation. Only 20 cases within the ASV-R cohort (9.6%) were managed with isolated ablation of the ASV alone, extending up to its confluence with the GSV, while the GSV remained patent. In 95 limbs of the ASV-R group (45.7%), no GSV reflux was present, and GSV ablation was therefore limited to only a few centimeters distal to the saphenofemoral junction. In contrast, in the majority of limbs (113 legs; 54.3%), additional segmental reflux of the GSV was present further distally. In these cases, complete ablation of the GSV was consistently performed in addition to ASV ablation, covering the full extent of the incompetent segment. In the GSV-R group, the non-refluxive ASV or an existing posterior accessory saphenous vein (PASV) was ablated via a separate access in 149 cases (58.2%).

As shown in Table 1, significantly more treatments were performed in the ASV-R group using the thinner 4-French catheter, which is easier to position in shorter venous segments.

In the ASV-R cohort, concomitant sclerotherapy or phlebectomy of varicose tributaries was performed in 13 patients (6.3%). In the GSV-R cohort, simultaneous treatment of varicose tributaries by phlebectomy or foam sclerotherapy was performed in 14 cases (5.5%).

Postinterventional clinical results and ultrasound examinations were collected in all cases except for three in the ASV-R cohort. In the ASV-R group, first postoperative follow-up revealed insufficient occlusion with persistent reflux in 4 limbs (1.9%). All of these cases underwent redo ablation within a short period of time. These cases continued to be observed for subsequent outcome analyses, including recurrence rates and the need for post-ablation sclerotherapy. In contrast, in the GSV-R cohort, reflux was successfully eliminated in all cases and no early thermal redo procedures were required (*p* = 0.04).

### 3.3. Postinterventional Complications

No major complications were observed in either cohort. Minor complications requiring re-presentation and, if necessary, medical or physical treatment—but no further interventional procedures—did not differ significantly between groups (Table 2).

In the ASV-R cohort, 11 minor complications (5.3%) were recorded: five cases of symptomatic phlebitis, two hematomas, two sensory disturbances, one mild allergic reaction, and one case of thrombophlebitis at the antecubital venous access site used for sedation.

In the GSV-R cohort, 11 minor complications (4.3%) occurred. These included three patients with endothermal heat-induced thrombosis (EHIT) > 1 who required follow-up examinations and anticoagulation, and three patients with postoperative thoracic symptoms who underwent further diagnostic evaluation, all of which were negative for pulmonary embolism or cardiac events. Two patients developed thrombophlebitis, only treated symptomatically. In one case, postoperative bleeding at a puncture incision required local management. One patient experienced a transient, thermally induced, non-necrotic skin irritation, and one patient developed transient lymphedema requiring physical therapy (Table 2).

### 3.4. Secondary Interventions Due to Recurrence

In both cohorts, patients regularly returned for follow-up, which was explicitly encouraged by the treating team. Follow-up examinations were offered regardless of whether recurrent or persistent venous symptoms were present; however, patients were advised to return promptly if symptoms occurred.

**Figure 1:**
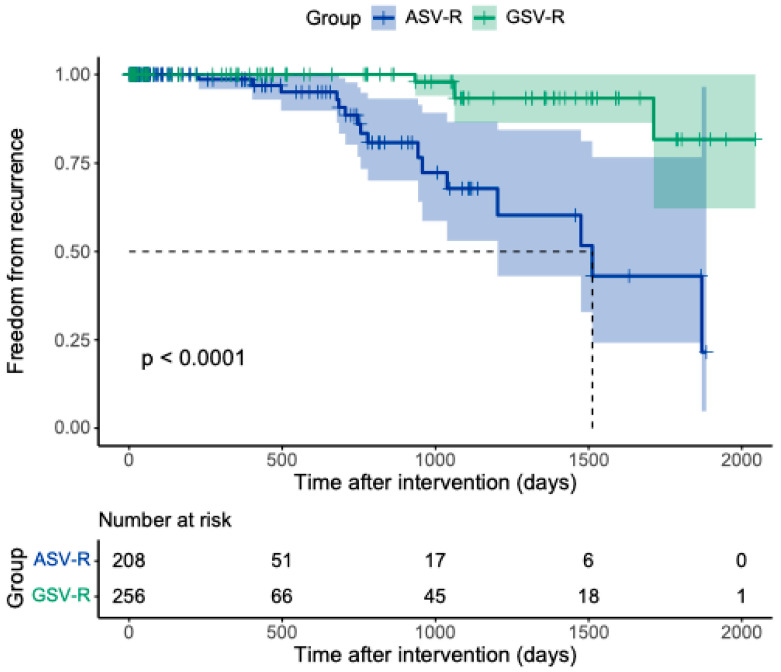
Kaplan-Meier curve showing freedom from recurrence after interventions for the GSV-R cohort and ASV-R cohort. The ASV-R Cohort exhibited a higher risk of recurrence compared with the GSV-R Cohort. The shaded areas represent 95% confidence intervals, and the *p*-value from the log-rank test is indicated on the plot.

In the ASV-R group, 16 cases required redo laser ablation due to recurrence. For our analysis, any new reflux in venous structures attributable to the GSV was considered a recurrence, even if the ASV, rather than the GSV, was the primary reflux-conducting vein. Accordingly, in 7 of the 16 recurrence cases (43.8%) in the ASV-R group, the recurrence involved the GSV territory. In the GSV-R cohort, recurrences were exclusively distal refluxing segments of the GSV. To date, no proximal junctional recurrences or recurrences attributable to varicose transformation of the ASV have been observed in this group. Kaplan–Meier analysis demonstrated a clear separation of recurrence-free survival curves between cohorts. In univariate Cox regression, ASV-R was associated with a markedly increased hazard of recurrence compared with GSV-R (HR 8.41, 95% CI 2.78–25.4, *p* < 0.001). Figure 1 illustrates the survival curve with recurrence-free survival plotted on the y-axis.

Follow-up duration was right-skewed in both cohorts, with a small number of long-term observations. Therefore, follow-up time among censored observations was summarized using medians and interquartile ranges (IQR) and compared between cohorts using a non-parametric Mann–Whitney U test. Median follow-up among censored observations was 36.5 days (IQR 11.25–391.25) in the ASV-R cohort and 14 days (IQR 10–468.75) in the GSV-R cohort, with no statistically significant difference between cohorts (*p* = 0.082).

No statistically significant difference was observed between the two cohorts regarding the frequency of subsequent sclerotherapy treatments. Post-ablation sclerotherapy was performed after 35 treatments in the ASV-R cohort and after 27 treatments in the GSV-R cohort. The difference did not reach statistical significance, as confirmed by the time-dependent Kaplan–Meier analysis.

### 3.5. Subgroup Analysis of the ASV-R Cohort

A subgroup analysis was conducted to assess whether concomitant segmental or long-segment GSV insufficiency in the presence of ASV insufficiency affected treatment outcomes. Clinically, cases with combined ASV and GSV insufficiency typically presented with a prominent tributary connection in the anteromedial thigh and frequently with pronounced varicosities of the medial calf. In contrast, patients with isolated ASV reflux without GSV involvement more commonly exhibited tributary varicosities located at the anterolateral thigh and lower leg.

**Figure 2:**
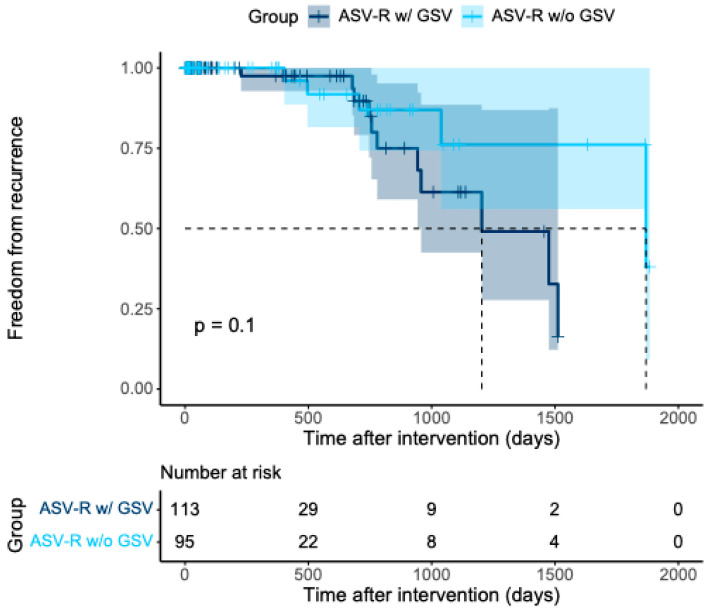
Kaplan–Meier curve illustrating freedom from recurrence in the ASV-R cohort (*n* = 208). Patients within the ASV-R group were stratified according to the presence of concomitant GSV incompetence (ASV-R w/ GSV; *n* = 113) versus absence of concomitant GSV incompetence (ASV-R w/o GSV; *n* = 95). Shaded areas represent the 95% confidence intervals.

Recurrence rates did not differ significantly between the two subgroups (Figure 2; *p* = 0.103; log-rank test). In the subgroup with isolated reflux of the ASV, without concomitant reflux of the GSV (ASV-R w/o GSV, *n* = 95 limbs), five cases of recurrence were observed. Of these, four were proximal recurrences attributable to the ASV territory, and one case showed insufficiency of the GSV. In limbs with concomitant reflux of the GSV (ASV-R w/ GSV, *n* = 113), recurrences requiring further intervention were diagnosed 11 times. In four cases, these represented distal recurrences of the GSV trunk, whereas in the remaining seven cases, proximal recurrence formations in the groin region were observed, all maintaining a venous connection to the femoral vein.

**Figure 3:**
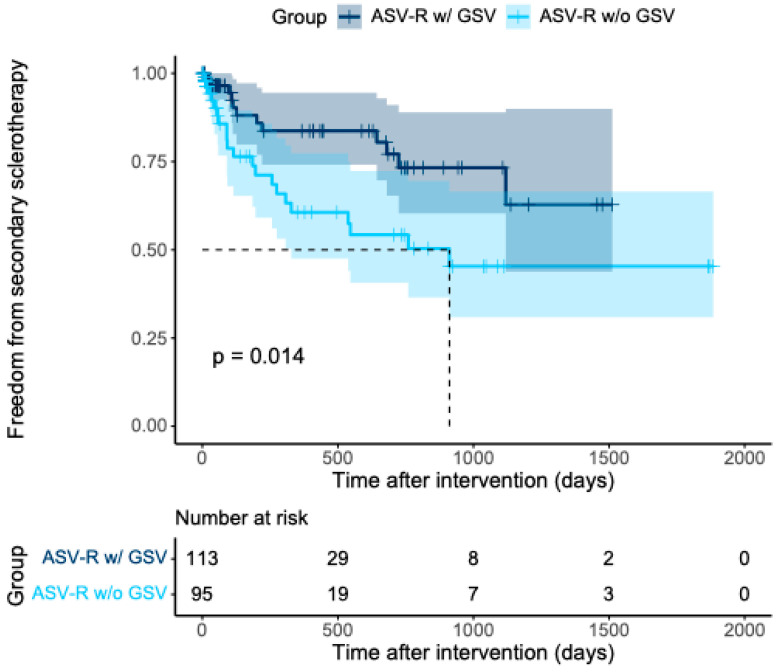
Kaplan–Meier analysis illustrating the incidence of subsequent sclerotherapy within the ASV-R cohort, stratified according to the presence of concomitant GSV incompetence (ASV-R w/ GSV; *n* = 113) versus absence of GSV incompetence (ASV-R w/o GSV; *n* = 95). Shaded areas represent the 95% confidence intervals.

Notably, a statistically significant difference was observed with respect to the frequency of subsequent sclerotherapy. Patients with isolated ASV reflux required additional sclerotherapy of tributary veins significantly more often (Figure 3; *p* = 0.014; log-rank test).

## 4. Discussion

In our study, EVLA for great saphenous vein reflux (GSV-R) demonstrated favorable outcomes, with a 100% technical success rate and a low complication profile, including a 1.2% incidence of EHIT > 1, which is comparable to previously published data [18,19]. By contrast, we interpret the slightly lower early technical success rate observed in the ASV-R cohort (98.1%) together with the more frequent use of the 4-French fiber, as being due to the increased technical difficulty of puncturing and cannulating a considerably shorter truncal vein [3,7,12].

Taken together with the higher recurrence rates observed in the ASV-R group, these findings suggest that EVLA for ASV insufficiency is technically more demanding than treatment of axial GSV reflux. Importantly, both cohorts were assembled consecutively, without case selection, reflecting routine clinical practice. From a clinical perspective, this underlines the importance of transparent preoperative patient counseling in cases of challenging anatomy, as well as the need for organizational readiness to provide redo interventions, which may not be separately reimbursed depending on the healthcare system. Another study similarly reported the need for redo interventions with thermal ablation, including laser and radiofrequency, following unsuccessful initial procedures, at a frequency comparable to that observed in our cohort [12].

To date, only limited data on thermal ablation for ASV reflux are available in the literature, and existing studies differ substantially regarding indications, prevalence, and technical approaches, limiting direct comparability. In a registry-based study by Deol et al., 103 thermal ablations for ASV reflux were identified and compared with 10,371 GSV treatments performed during the same period, resulting in a proportion of only 1% ASV interventions [15]. This is considerably lower than previously reported rates and also markedly lower than in our cohort. During the recruitment period from April to December 2019, we performed 22 ablations for cases with ASV reflux compared with 256 procedures for GSV reflux, corresponding to a proportion of 8.6%.

In an earlier study by Theivacumar et al., outcomes of 33 ASV treatments were reported, of which 12 were performed for recurrent disease following surgical or endovenous GSV treatment. A minimum straight vein length of 10 cm distal to the saphenofemoral junction was required for inclusion [14]. Such an anatomical restriction was not applied in our cohort, as the use of radially emitting laser fibers allowed effective ablation of considerably shorter straight venous segments.

In the majority of ASV-R cases without additional distal GSV reflux, ablation of the incompetent ASV was combined with ablation of the proximal GSV. This treatment strategy is practiced in several German vein centers and is based on the concept that terminal valve insufficiency with ASV reflux is frequently accompanied by dilation of the proximal GSV, potentially predisposing to later GSV insufficiency and recurrence [20]. Similarly to surgical high ligation, flush ablation of the proximal GSV can theoretically reduce this risk. It may be worth discussing whether the subsequent development of GSV insufficiency after successful ASV treatment should be classified as a recurrence or rather as progression of the disease. Based on the available data, no definitive conclusions can be drawn regarding the effectiveness of this strategy in preventing recurrences.

The optimal management of varicose tributaries in conjunction with truncal vein ablation remains a subject of ongoing discussion [21,22,23,24]. Adjunctive treatment of side branches was rarely applied in either group in our cohort (6.3% in ASV-R and 5.5% in GSV-R). The predominant use of a staged approach, rather than combined treatments, reflects clinical experience that dependent tributaries regress spontaneously in many cases, with secondary sclerotherapy reserved for persistent symptomatic or cosmetically relevant varicosities. Our data suggest, that the typically present anterolateral tributaries associated with isolated ASV reflux may regress less completely than the anteromedial tributaries which connect both truncal veins in combined ASV and GSV reflux. Further studies are warranted to better characterize these patterns and to support individualized, patient-specific treatment planning.

Furthermore, in light of the comparatively higher recurrence rate observed in the ASV-R cohort, it is worth discussing techniques that could potentially result in more effective ablations, lower recurrence rates, or reduced need for subsequent sclerotherapy. For example, extended laser ablation, including more extensive treatment of meandering superficial tributaries in addition to the truncal veins, could potentially achieve a more complete varicose vein reduction and improved outcomes [25]. At the same time, the anatomical and technical limitations of endovenous ablation procedures must be acknowledged. In this context, modern open varicose vein surgery with flush ligation and stripping, which is technically feasible for both GSV and ASV insufficiency, may represent a valid alternative treatment option. Moreover, despite the increasing adoption of endovenous treatment strategies, favorable outcomes with low perioperative morbidity have continued to be observed for open surgical procedures in specialized centers [26,27]. However, no sufficient data are available to substantiate these approaches, particularly regarding a direct comparison of both procedures in ASV insufficiency.

Several limitations of this study must be acknowledged. The present analysis is a single-center, retrospective observational study based on medical records and includes procedures performed by a single surgeon, which may limit generalizability and introduce potential bias. A key limitation is the low number of recurrence events in the GSV-R cohort, which precludes a power-optimized comparative design and limits the ability to detect moderate differences between groups. The size of the ASV-R cohort was clinically constrained, and further expansion of the comparison cohort would not necessarily have resulted in a meaningful gain in information due to the low event rate.

Furthermore, the cohorts were derived from different calendar periods and exhibited modest differences in follow-up duration. To address this, a time-to-event analytical framework was applied, which appropriately accounts for variable follow-up and censoring. Nevertheless, residual confounding related to temporal factors cannot be fully excluded and should be considered when interpreting the magnitude of the observed effect. Given the shorter follow-up in the ASV-R cohort, the observed recurrence risk may be underestimated.

From a methodological perspective, however, it remains uncertain which alternative study design would be better suited to address these challenges under routine clinical conditions. In general, the organization and adherence to follow-up examinations after venous interventions are demanding for both healthcare providers and patients, and the realization of extended follow-up periods—even in prospective studies—remains inherently uncertain. Notable strengths of the study include its consecutive design and comparative approach, which provide valuable real-world insights into the feasibility and clinical outcomes of endovenous laser ablation for ASV reflux in routine practice. Importantly, the large observed effect size and consistency across analyses suggest that differences in follow-up duration alone are unlikely to account for the results.

## 5. Conclusions

In this retrospective cohort study based on consecutive, unselected routine treatment data, EVLA of ASV reflux proved to be technically more demanding than treatment of axial GSV reflux. Time-to-event analyses indicated a higher recurrence risk in the ASV-R cohort, highlighting the need for careful patient counseling and organizational readiness for potential redo interventions. Further prospective and comparative studies are required to better define the effectiveness of thermal ablation strategies for anterior saphenous vein insufficiency and to support patient-centered, individualized treatment decisions.

## Figures and Tables

**Table 1:** Baseline characteristics.

Parameter	ASV-R	GSV-R	*p*-Value
Number of patients	185	197	
Sex			<0.001
Male	36	73	
Female	149	124	
Age; years; mean (±SD)	47.2 (±14.7)	51.8 (±15.6)	0.004
Body mass index; kg/m^2^; median (range)	25.55 (19.3–47.5) ^a^	24.25 (17.7–52.2) ^b^	0.002
Number of treated limbs	208	256	
CEAP			<0.001
C2	158	139	
C3	45	91	
C4	5	24	
C5	0	0	
C6	0	2	
C3–6	50	117	
Side			0.652
Left	110	130	
Right	98	126	
Max. diameter of leading refluxive vein; mm; median (range)			0.087
	7 (3–22) ^c^	8 (5–18)	
Ablation length of the main reflux vein; cm; median (range)			<0.001
	15 (2–45) ^d^	45 (25–62)	
Catheter type			<0.001
4 French, 400 µm core fiber	70	49	
6 French, 600 µm core fiber	138	207	
Sedation			0.098
Yes	126	174	
No	82	82	

SD: standard deviation; CEAP: clinical, etiological, anatomical, and pathophysiological classification. ^a^: data lacking in 17 individuals; ^b^: data lacking in 19 individuals; ^c^: data lacking in 18 cases; ^d^: data lacking in 21 cases.

**Table 2:** Complications after intervention.

Parameter	ASV-R	GSV-R	*p*-Value
Number of patients	185	197	
Number of treated limbs	208	256	
Major complications	0	0	
Minor complications	11	11	0.617
EHIT > 1	0	3	
Symptomatic phlebitis	5	2	
Bleeding/hematoma	2	1	
Sensory disorders	2	0	
Skin irritation	0	1	
Lymphedema	0	1	
Local allergic reaction	1	0	
Thrombophlebitis (Arm)	1	0	
Nonspecific thoracic symptoms	0	3	

EHIT: endothermal heat-induced thrombosis.

## Data Availability

The raw data supporting the conclusions of this article will be made available by the authors on request.
